# Six-Membered Aromatic Polyazides: Synthesis and Application

**DOI:** 10.3390/molecules201019142

**Published:** 2015-10-21

**Authors:** Sergei V. Chapyshev

**Affiliations:** Institute of Problems of Chemical Physics, Russian Academy of Sciences, Chernogolovka 142432, Moscow Region, Russian; E-Mail: s.chapyshev@mail.ru; Tel.: +7-4965-223-507

**Keywords:** polyazides, high-energy materials, high-spin nitrenes, cross-linking agents, photoresists, click-reactions

## Abstract

Aromatic polyazides are widely used as starting materials in organic synthesis and photochemical studies, as well as photoresists in microelectronics and as cross-linking agents in polymer chemistry. Some aromatic polyazides possess high antitumor activity, while many others are of considerable interest as high-energy materials and precursors of high-spin nitrenes and C_3_N_4_ carbon nitride nanomaterials. The use of aromatic polyazides in click-reactions may be a new promising direction in the design of various supramolecular systems possessing interesting chemical, physical and biological properties. This review is devoted to the synthesis, properties and applications of six-membered aromatic compounds containing three and more azido groups in the ring.

## 1. Introduction

To date, just about two dozen six-membered aromatic compounds containing three and more azido groups in the ring are known. Nevertheless, investigations of these polyazides have played an important role in the development of chemistry. Thus, the first data on the linear structure of the azido groups were obtained due to X-ray diffraction studies of crystalline 2,4,6-triazido-1,3,5-triazine [1,2,3,4,5]. The thermal and detonative decomposition of this triazide allowed as well the first preparation of diverse C_3_N_4_ carbon nitride nanomaterials and carbon nanotubes [6,7,8,9]. The first organic septet hexaradical was prepared by the photolysis of 1,3,5-triazido-2,4,6-tricyanobenzene [10]. Later, a great variety of high-spin nitrenes possessing unusual magnetic characteristics were obtained by the photolysis of various 1,3,5-triazidobenzenes, 2,4,6-triazidopyridines, 2,4,6-triazidopyrimidines and 2,4,6-triazido-1,3,5-triazine [11,12,13,14,15,16,17,18,19,20,21,22,23,24,25,26,27,28,29,30,31,32,33]. These studies of high-spin nitrenes opened up new prospects in design of new organic magnetic materials and provided important information about UV-vis, IR and EPR spectra of organic molecules with the ground quintet and septet spin-state. In particular, it was found that the placement of heavy bromine atoms in molecules of high-spin nitrenes can strengthen several times the magnetic properties of organic polyradicals [26,33]. Six-membered aromatic triazides have also played an important role in investigations of the reactivity of the azido groups. Thus, it was found that the more electron-deficient γ-azido groups of 2,4,6-triazidopyridines selectively react with electron-rich dipolarophiles, phosphines, phosphites and reductants [34,35]. By contrast, the more electron-rich α-azido groups of the same triazides are the most reactive toward electron-poor dipolarophiles. Since 2002, organic azides are widely used as starting materials in the copper-catalyzed azide-alkyne cycloadditions or so-called click-reactions to prepare organic compounds with interesting chemical, physical and biological properties [36,37]. Recent examples on the use of aromatic diazides in such reactions demonstrate a huge synthetic potential of aromatic polyazides in design of new polyfunctional organic materials [38,39,40,41,42,43,44,45,46,47,48,49,50,51,52,53,54,55,56,57,58,59,60]. The present review provides concise and precise updates on the latest progress in the synthesis and investigation of six-membered aromatic polyazides made by July 2015.

## 2. Cyanuric Triazide

**Scheme 1:**
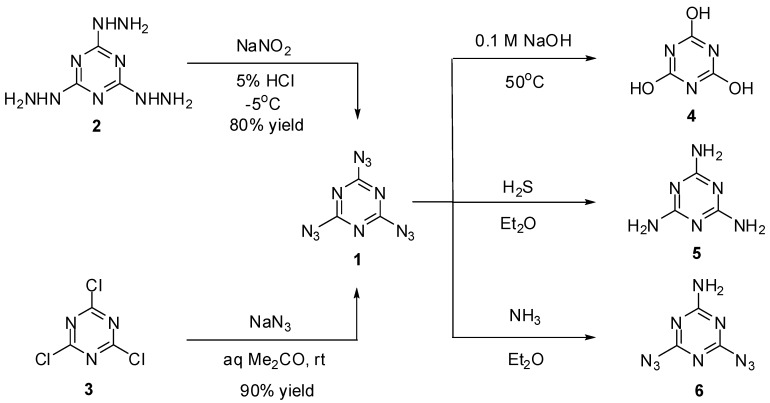
Synthesis and some transformations of triazide **1**.

2,4,6-Triazido-1,3,5-triazine or cyanuric triazide (**1**) was prepared for the first time by H. Finger in 1907, using diazotization of *tris*-hydrazine **2** with NaNO_2_ in acidic media (Scheme 1) [61]. Several years later, a more convenient way to prepare **1** based on triazidation of cyanuric chloride **3** with sodium azide in aqueous acetone was developed by Ott and Ohse [62]. Currently, triazide **1** is readily prepared in almost quantitative yield by addition of small portions of solid sodium azide to solutions of chloride **3** in acetone at room temperature [63,64]. On crystallization from ethanol, triazide **1** forms dense colorless crystals with a melting point of 94 °C [61,62,63,64]. Due to its high nitrogen content (82.35%), triazide **1** is very sensitive to impact, friction and electrostatic discharge. On working with it, one should always handle **1** only with plastic spatulas and use thick gloves behind a blast shield.

**Scheme 2:**
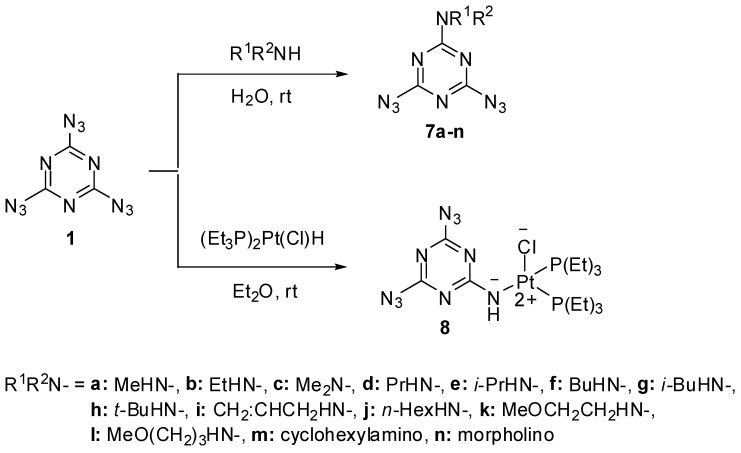
Reactions of triazide **1** with alkyl amines and (Et_3_P)_2_Pt(Cl)H.

Early studies have shown that triazide **1** was quickly hydrolyzed to cyanuric acid (**4**) on heating in 0.1 M solutions of sodium hydroxide at 50 °C (Scheme 2) [62]. By action of gaseous hydrogen sulfide, triazide **1** was reduced to melamine (**5**) [65]. Similar reaction of **1** with gaseous ammonia afforded aminodiazide **6** in almost quantitative yield [65]. In the last case, the reaction can formally be considered as nucleophilic substitution reaction of the azido group in **1** for the amino group. Thus, this triazide reacts in the same manner with various alkyl amines to give aminodiazides **7a**–**n** in high yields [66]. All diazides **5** and **7a**–**n** present as white solids with a melting point ranging from 44 °C for **7c** till 200 °C for **6** and are almost insensitive to impact, friction and electrostatic discharge. These compounds may be of practical interest as insensitive high-energy additives and reactants for click-reactions. The reduction of triazide **1** with hydridoplatinum complex (Et_3_P)_2_Pt(Cl)H gives air-stable amidoplatinum complex **8** in 100% yield (Scheme 2) [67]. The latter can be considered as an analog of aminodiazides **6** and **7a**–**n**.

**Scheme 3:**
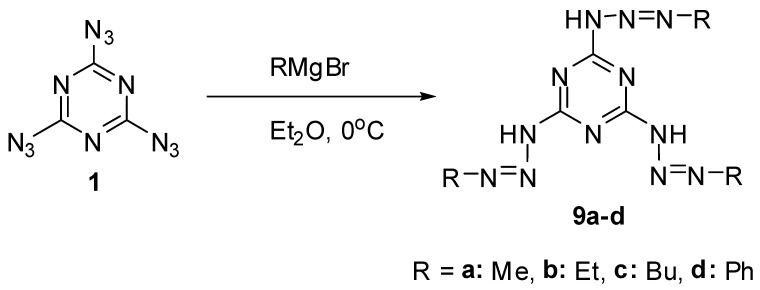
Reactions of triazide **1** with Grignard reagents.

All three azido groups of **1** react with Grignard reagents to give *tris*-triazenes **9a**–**d** in 47%–70% yield (Scheme 3) [68]. Triazenes are of considerable interest as anticancer drugs and powerful carcinogens [69,70,71].

**Scheme 4:**
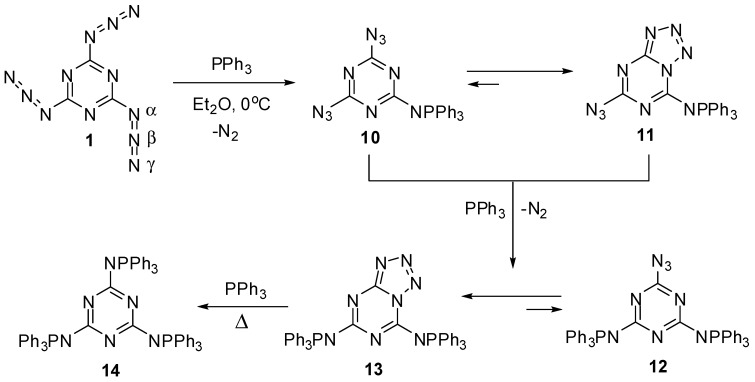
Reactions of triazide **1** with triphenylphosphine.

The first attempt to investigate the Staudinger-Meyer reaction of triazide **1** was undertaken by Kesting in 1923 [72]. The reaction of this triazide with an excess of triphenylphosphine in ether gave a single product that was assigned to azide **12**, based on the elemental analysis data (Scheme 4). However, recent studies showed that this product really corresponds to tetrazole **13** [63,64]. The first stage of the reaction involves the formation of diazide **10** that exists in organic solutions in equilibrium with azidotetrazole **11**. The ratio of **10** to **11** in chloroform and dimethylsulfoxide solutions is ~1:3 and 1:50, respectively [64]. The addition of two equivalents of triphenylphosphine to triazide **1** gives tetrazole **13** as a single product. The structures of tetrazoles **11** and **13** were unambiguously proved by using ^13^C-, ^15^N- and ^31^P-NMR spectroscopy and X-ray diffraction analysis [63,64]. Tetrazole **13** does not react with triphenylphosphine in solution. The *tris*-adduct **14** can only be obtained by melting **13** with an excess of triphenylphosphine [64].

In contrast to azides **10** and **12** containing strong electron-donating aza-ylide substituents on the triazine ring, triazide **1** itself does not isomerize to tetrazolo[5,1-*a*]-[1,3,5]triazines in organic solutions due to insufficiently high nucleophilicity of the *endo*-cyclic nitrogen atoms [64]. This triazide has become the first organic azide characterized with X-ray diffraction analysis [1,2,3,4,5]. These structural studies proved that organic azides have a linear structure of the azido groups. The molecule of **1** is planar with bent azido groups giving rise to *C*_3h_ symmetry (Scheme 4). The azido groups show π bond localization with a short Nβ–Nγ bond distance of 1.1156 Å (bond order ≈ 2.5) and a longer Nα–Nβ bond distance of 1.2658 Å (bond order ≈ 1.5) [63]. The C–Nα–Nβ angle is 112.5°, and the Nα–Nβ–Nγ angle is slightly bent at 172.0°. The distance between the layers is 2.947 Å, and the crystal density is 1.736 g/cm^3^ [63]. In IR spectra, the azido groups of **1** show four strong bands at ν 2192, 2158, 2115 and 1198 cm^−1^ [63]. The ^13^C-NMR spectrum of **1** in acetone-*d*_6_ displays a signal of the carbon atoms at δ 171.9 ppm [63]. The ^15^N-NMR spectrum of **1** in CDCl_3_ shows four signals of the nitrogen atoms at δ −131.3 (Nγ), −142.6 (Nβ), −160.2 (N-ring) and −257.3 (Nα) ppm [73].

In material chemistry, triazide **1** is often used as a reference compound in studies of new high-energy nitrogen-rich organic compounds [74,75,76,77,78,79,80]. Its sensitivity to impact, friction and spark is estimated as 6.2 cm (H_50_), <0.5 kg and <0.36 J, respectively [77]. This triazide has also one of the highest positive heats of formation (Δ*H*_f_ = 1053 kJ/mol) among aromatic polyazides [78]. The thermal decomposition and detonation of **1** are used nowadays for preparation of C_3_N_4_ and C_3_N_5_ carbon nitrides and carbon nanotubes [6,7,8,9]. These materials possess unique mechanical, physical and chemical properties and are of considerable interest for industrial applications.

**Scheme 5:**
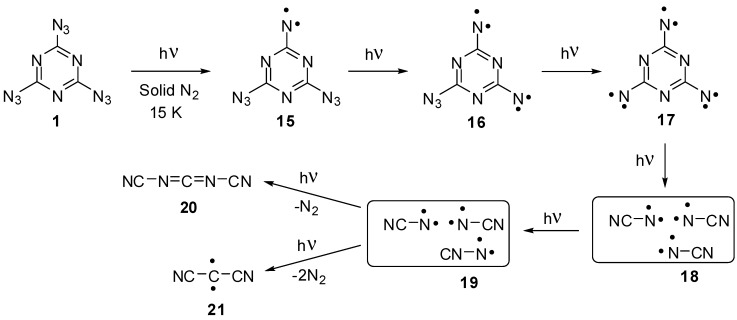
Photolysis of triazide **1**.

The first attempt to investigate the photochemistry of triazide **1** was undertaken by Moriarty and co-workers in 1966 [81]. It was found that low-temperature photolysis of crystalline **1** produces the stable at room temperature triplet nitrene **15** showing the largest zero-field splitting (zfs) parameter of |*D*| = 1.445 cm^−1^ among all triplet arylnitrenes (Scheme 5). More recent studies of the photolysis of crystalline **1** at 4 K allowed the EPR detection of quintet dinitrene **16** with magnetic parameters |*D*| = 0.28 cm^−1^ and |*E*| = 0.058 cm^−1^, rather unusual for quintet dinitrenes [82]. A few years later, Sato and colleagues succeeded in EPR detection of septet trinitrene **17** formed during the photolysis of triazide **1** in solid nitrogen matrices [14,15]. It was found that three *endo*-cyclic nitrogen atoms of the triazine ring favor the extremely high localization of spins on the nitrene units of trinitrene **17**. As a result, this trinitrene showed the largest negative value of *D* = −0.123 cm^−1^ among all organic septet hexaradicals. Trinitrene **17** was photochemically unstable and decomposed to form triplet cyanonitrenes **18**, that underwent further photochemical rearrangements to form triplet isocyanonitrene **19**, dicyanocarbodiimide **20** and triplet dicyanocarbene **21**. Overall, the photochemical studies showed that trinitrene **17** generated by the photolysis of triazide **1** possesses the record value of magnetic anisotropy among all organic hexaradicals, but is extremely reactive and photochemically unstable. Its decomposition leads to carbodiimide **20**, similarly to the formation of C_3_N_4_ carbon nitrides during the thermal and detonative decomposition of triazide **1**.

## 3. Triazidopyrimidines

**Scheme 6:**
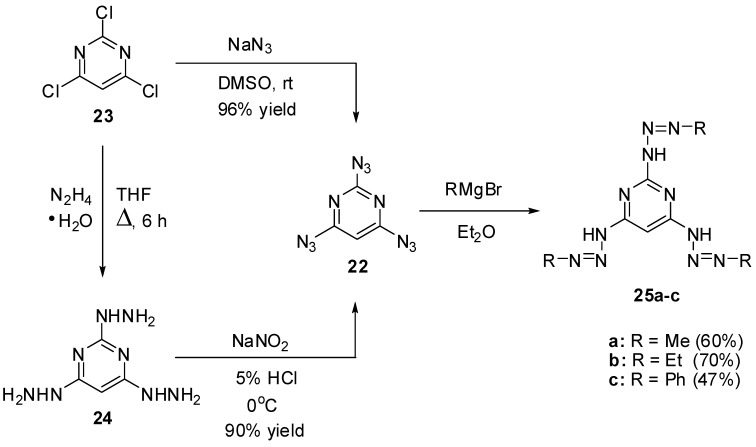
Synthesis and reactions of triazide **22** with Grignard reagents.

At the moment, there are known just five triazidopyrimidines. The first of them, 2,4,6-triazidopyrimidine (**22**), was prepared by Pochinok and co-workers in 1979 (Scheme 6) [68]. The azidation of commercially available trichloride **23** with excess sodium azide in dimethylsulfoxide at room temperature gave triazide **22** in 96% yield. On the other hand, the diazotization of *tris*-hydrazine **24** with NaNO_2_ in acidic media afforded triazide **22** in 90% yield. This triazide was used as a starting material to prepare *tris*-triazenes **25a**–**c** (Scheme 6) [68]. Recently, triazide **22** was obtained by azidation of trichloride **23** with excess sodium azide in acetone at room temperature in 91% yield [78].

**Scheme 7:**
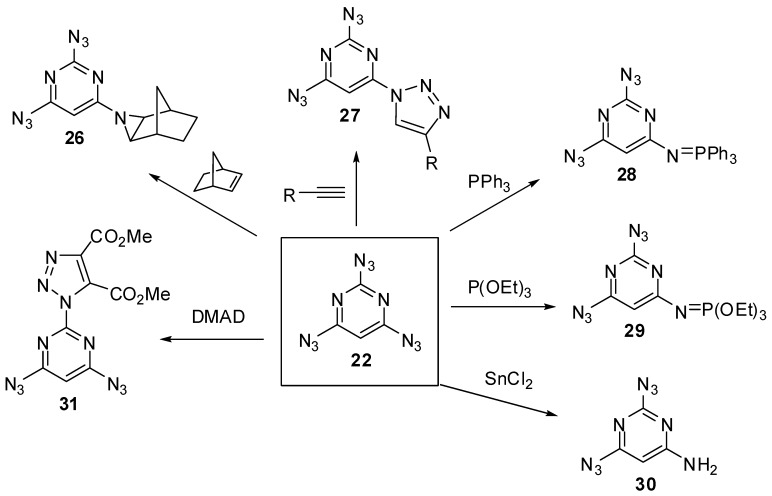
Theoretically predicted reactions for triazide **22**.

On crystallization from ethanol, triazide **22** forms dense colorless crystals with a melting point of 98 °C [68,78]. In comparison with cyanuric triazide, triazide **22** is much less sensitive to impact and friction. It may explode only under a very strong blow. Nevertheless, on working with **22**, it is better to handle it only with plastic spatulas and use thick gloves behind a blast shield. In IR spectra, the azido groups of **22** show two weak bands at ν 2177 and 2100 cm^−1^ and two bands of medium intensity at ν 2144 and 1161 cm^−1^ [78]. The ^1^H-NMR spectrum of **22** in CDCl_3_ shows a signal of the proton at δ 6.88 ppm [73]. The ^13^C-NMR spectrum of **22** in CDCl_3_ displays three signals of the carbons at δ 94.3 (C-5), 161.5 (C-2) and 164.9 (C-4, C-6) ppm [73]. The ^15^N-NMR spectrum of **22** in CDCl_3_ shows seven signals of the nitrogen atoms at δ −134.5 (Nγ, 4,6-N_3_), −135.1 (Nγ, 2-N_3_), −140.0 (Nβ, 2-N_3_), −141.0 (Nβ, 4,6-N_3_), −145.6 (*N*-ring), −262.7 (Nα, 2-N_3_) and −264.8 (Nα, 4,6-N_3_) ppm [73]. The ^13^C- and ^15^N-NMR spectra can be very helpful in predicting the chemical reactivity of nonequivalent azido groups of 2,4,6-triazidopyrimidines. Thus, quantum-chemical studies showed that the azido groups in positions 4 and 6 of triazide **22** are the most electron-deficient [73]. In ^15^N-NMR spectra, these groups display the most shielded signal of the Nα atoms and should selectively react with electron-rich dipolarophiles, phosphines, phosphites and reductants to give diazides **26**–**30** (Scheme 7) [73]. On the other hand, the reaction of **22** with electron-deficient dimethyl acetylenedicarboxylate (DMAD) should selectively occur on the electron-rich azido group in position 2 of the pyrimidine ring to give adduct **31**.

**Scheme 8:**
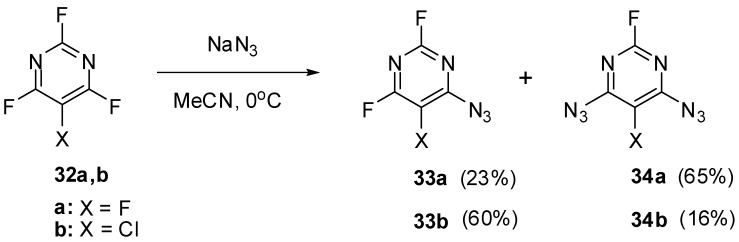
Mono- and diazidation of fluorides **32a**,**b**.

The high electron-deficiency of the azido groups in positions 4 and 6 of triazide **22** is also manifested in the most deshielded signals of the C-4 and C-6 atoms in the ^13^C-NMR spectrum of this triazide. As a rule, the first stage of azidation of 2,4,6-trihalopyrimidines is the replacement of the halogen atom for the azido group in position 4 of the pyrimidine ring. Thus, trihalides **32a**,**b** reacted with sodium azide in actetonitrile at room temperature to give only monoazides **33a**,**b** and diazides **34a**,**b** (Scheme 8) [83].

**Scheme 9:**
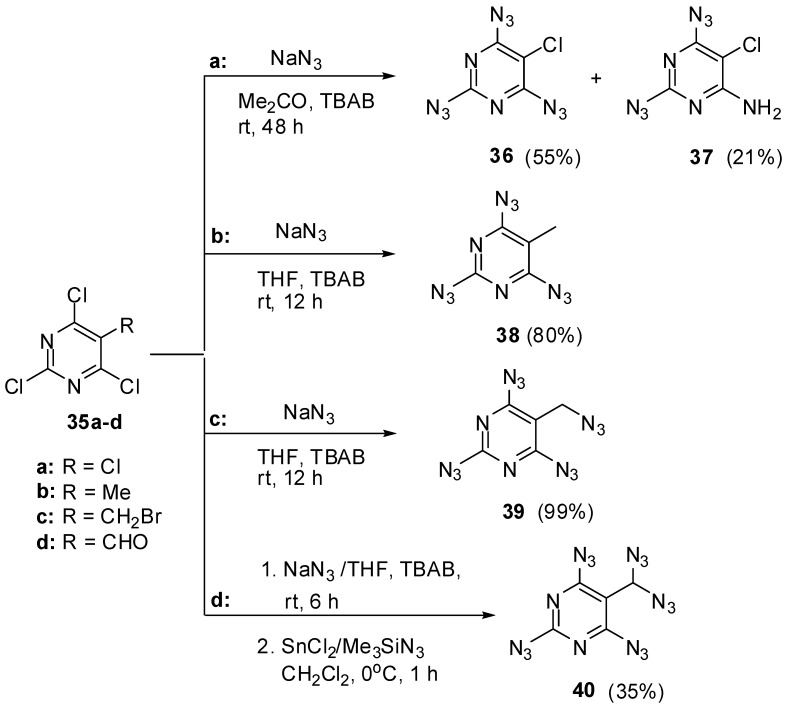
Synthesis of triazides **36**, **38**, **39** and **41**.

Similarly to triazide **22**, four other 2,4,6-triazidopyrimidines were synthesized (Scheme 9) [78]. The azidation of tetrachloride **35a** with sodium azide in acetone at room temperature in the presence of *tert*-butylammonium bromide (TBAB) gave a mixture of triazide **36** and diazide **37** in 55% and 21% yields, respectively. The reactions of trichlorides **35b**,**c** with sodium azide in tetrahydrofuran (THF) at room temperature in the presence of TBAB gave triazide **38** and tetraazide **39** in 80% and 99% yields, respectively. Finally, the reaction of trichloride **35d** with sodium azide in THF at room temperature in the presence of TBAB followed by addition of stannous chloride and trimethylsilylazide (TMSA) to intermediate products gave pentaazide **40** in 35% yield. Despite the high-nitrogen content, tetraazide **39** and pentaazide **40** are not very sensitive to impact and friction since their low melting points (22.5 and −48 °C, respectively) [78].

The structure of triazide **38** was studied by X-ray diffraction analysis [78]. It was found that the least electron-deficient azido group in position 2 of **38** has a somewhat elongated C–Nα bond distance (1.412 Å), a shortened Nα–Nβ bond distance (1.251 Å) and the least bent Nβ–Nβ–Nγ angle (172.9°). Similar parameters for the azido group in position 4 of **38** equal to 1.406 Å, 1.257 Å and 172.4°, respectively. At the same time, all three azido groups of **38** have the same Nβ–Nγ bond distances (1.119 Å) and nearly the same C–Nα–Nβ angles (~113.5°). The more bent Nα–Nβ–Nγ angles and more elongated Nα–Nβ bond distances in the azido groups in positions 4 and 6 of **38** suggest that these groups should be the least stable during thermolysis and decompose first to form triplet nitrenes and then amines.

Triazides **22**, **38**, **39** and **40** are typical high-energy nitrogen-rich compounds, which thermal and detonative decomposition is used for preparation of C_3_N_4_ carbon nitrides and carbon nanotubes [78]. Triazide **22** is also of interest as an antitumor agent showing high activity against Sarcoma 180, Piss lymphosarcoma and Guerin carcinoma, along with low toxicity (>1000 mg/kg) [84].

**Scheme 10:**
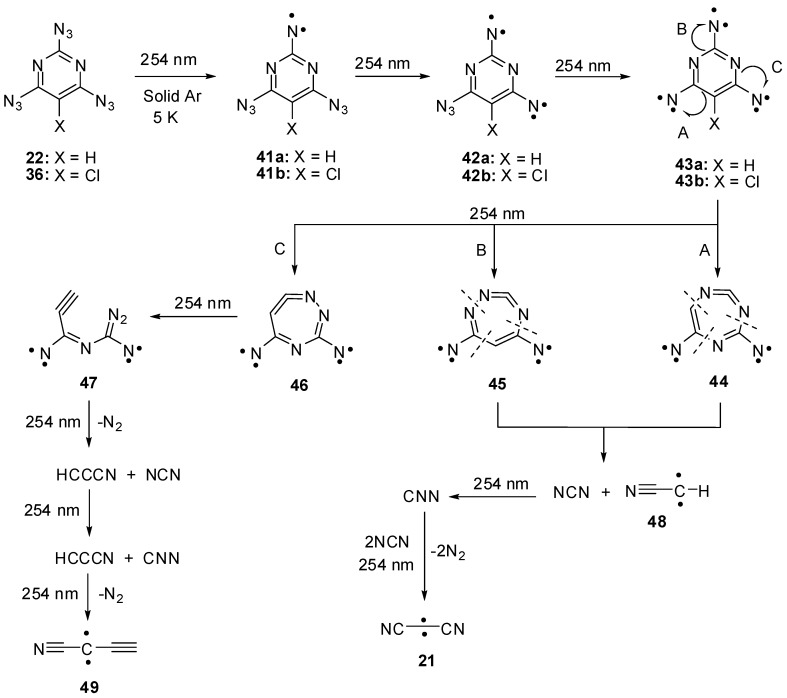
Photolysis of triazides **22** and **36**.

Triazides **22** and **36** were used as starting materials in several photochemical studies. First attempts to detect nitrenes during the photolysis of triazides **22** and **36** in frozen organic solutions at 77 K were unsuccessful [85]. Recently, the photolysis of **22** and **36** was studied in argon matrices at 5 K [30]. Using this technique, it was found that photochemical decompositions of **22** and **36** occur selectively to subsequently give triplet nitrenes **41a**,**b**, quintet dinitrenes **42a**,**b** and septet trinitrenes **43a**,**b** (Scheme 10). Since the presence of only two *endo*-cyclic nitrogen atoms in the ring, trinitrene **43a** showed *D* = −0.1122 cm^−1^ that was by ~10% smaller than the *D*-value of trinitrene **17**. As in the case of trinitrene **17**, trinitrenes **43a**,**b** were photochemically unstable and decomposed to form triplet nitrenes NCN and NNC as well as triplet carbenes NCCCN, HCCN and HCCCCN. Nitrenes NCN and NNC and carbene NCCCN were previously also observed during the photolysis of cyanuric triazide [15]. The formation of the same reactive intermediates during the photolysis of triazides **17** and **22** explained well the formation of the same C_3_N_4_ carbon nitrides and carbon nanotubes during thermal and detonative decomposition of both triazides. At the same time, the photolysis of triazide **22** additionally produces carbenes HCCN and HCCCCN that are typical only for decomposition of azidopyrimidines. Of these two carbenes, the last one has never previously been generated in a laboratory, but was known as a component of interstellar matter [86].

## 4. Triazidopyridazines

**Scheme 11:**
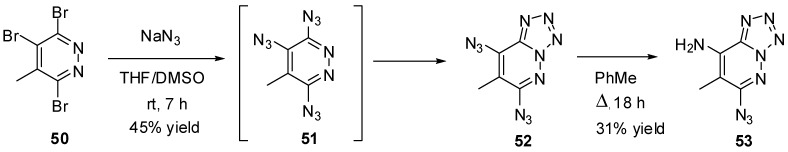
Triazidation of tribromide **50**.

Due to azide-tetrazole isomerization, triazidopyrazines and triazidopyridazines may exist only as short-living intermediates formed during the azidation of the corresponding trihalides. Thus, recent studies have shown that azidation of tribromide **50** with sodium azide gave diazidotetrazole **52** in 45% yield (Scheme 11) [87]. It was postulated that this tetrazole is formed from intermediate triazide **51**. The structure of **52** was confirmed by X-ray diffraction analysis. According to ^1^H-NMR studies, compound **52** exists in organic solutions exclusively as a tetrazole. On boiling in toluene, diazide **52** partially decomposed to form aminoazide **53** [87]. The latter also exists in organic solutions exclusively as a tetrazole.

## 5. Triazidopyridines

**Scheme 12:**
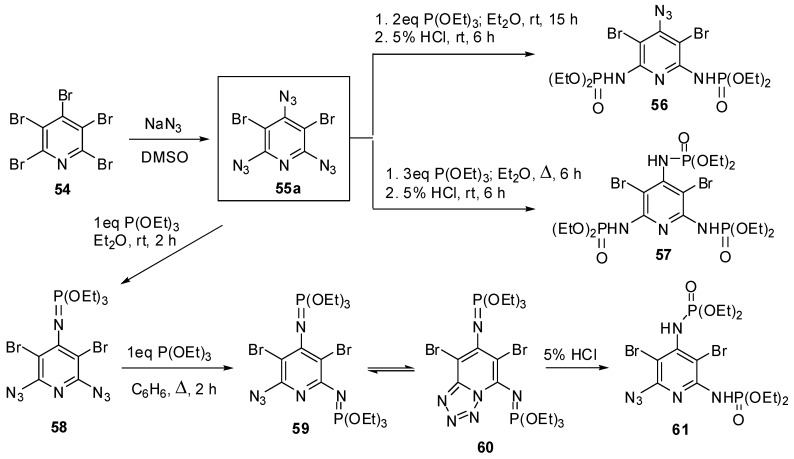
Synthesis and Staudinger-phosphite reactions of triazide **55a**.

2,4,6-Triazido-3,5-dibromopyridine (**55a**) was the first triazidopyridine, obtained by Moshchitskii and co-workers in 1979, using the reaction of pentabromopyridine (**54a**) with sodium azide (Scheme 12) [88]. The reaction was carried out in DMSO at room temperature for 15 h and gave triazide **55a** (a brown solid with a melting point 85–86 °C) in 97% yield. The reactions of **55a** with two and three molar equivalences of triethyl phosphite followed by acidic hydrolysis of the products with 5% HCl gave *bis*- and *tris*-adducts that were assigned to compounds **56** and **57**, respectively [88].

**Scheme 13:**
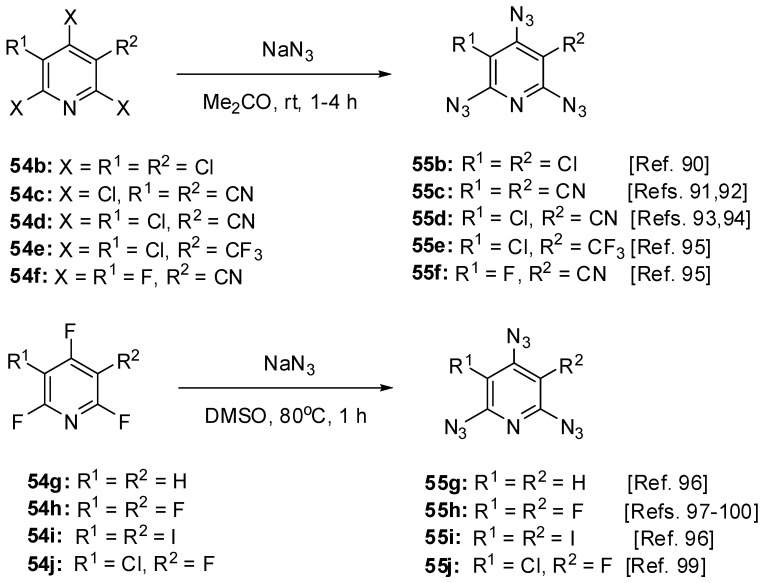
Synthesis of triazides **55b**–**j**.

Recently, triazide **55a** has been prepared by the reaction of **54a** with sodium azide in DMSO at 80 °C just for 30 min in 97% yield [89]. The addition of an equimolar amount of triethyl phosphite to **55a** resulted in the formation of diazide **58** as a single product (Scheme 12) [89]. The latter reacted with an equimolar amount of triethyl phosphite on boiling in benzene to form a mixture of compounds **59** and **60** in a nearly equal ratio. The hydrolysis of the mixture of **59** and **60** with 5% HCl afforded azide **61** as a single product [89]. The structures of compounds **58**–**60** were unambiguously confirmed by the data of ^1^H-, ^13^C- and ^31^P-NMR spectroscopic studies. According to these studies, the γ-azido group of triazide **55a** is the most electron-deficient and, as the result, the most reactive in the Staudinger reactions. After triazide **55a**, nine 2,4,6-triazidopyridines **55b**–**j** have been synthesized (Scheme 13) [90,91,92,93,94,95,96,97,98,99,100].

Owing to the presence of strong electron-withdrawing cyano- and trifluoromethyl-substituents on the pyridine ring, halides **54c**–**f** readily react with sodium azide in aqueous acetone at room temperature to form triazides **55c**–**f** in 86%–98% yields [91,92,93,94,95]. The least activated halide **54b** formed triazide **55b** (98%) on interaction with sodium azide in aqueous acetone only at temperature above 30 °C [90]. Similar reactions of trifluoropyridines **54g**–**j** with sodium azide in boiling aqueous acetone stopped, as a rule, on the stage of formation of the corresponding 2,4-diazidopyridines [97,98]. At the same time, fluorides **54g**–**j** readily underwent triazidation in hot DMSO to form triazides **55g**–**j** in 86%–96% yield [96,99]. In all cases, the first stage of the reaction was the replacement of the halogen atom in position 4 of the pyridine ring.

Triazides **55a**–**j** are solid materials (mp 63–135 °C) that are almost insensitive to impact, friction, and electrostatic discharge. Only triazides **55c** and **55g** may explode under a very strong blow [92,101]. In the IR spectra, the azido groups of **55a**–**j** show two strong bands in the ν 2180–2120 cm^−1^ region and a weak band about ν 2100 cm^−1^ [90,91,92,93,94,95,96,97,98,99,100]. In ^15^N-NMR spectra, the most electron-deficient γ-azido groups of **55a**–**j** display the most shielded Nα signals [73]. Thus, the ^15^N-NMR spectrum of triazide **55a** shows seven signals of the nitrogen atoms at δ −129.0 (*N*-ring), −134.8 (Nγ, α-N_3_), −139.8 (Nβ, α-N_3_), −140.8 (Nγ, γ-N_3_), −144.8 (Nβ, γ-N_3_), −265.8 (Nα, α-N_3_) and −276.2 ppm (Nα, γ-N_3_) [89]. The high electron-deficiency of the γ-azido groups of triazides **55a**–**j** is also manifested in the more shielded signals of the C-4 atoms in the ^13^C-NMR spectrum of these triazides. Thus, the ^13^C-NMR spectrum of triazide **55a** shows three signals of the carbon atoms at δ 98.2 (Cβ), 147.4 (Cγ) and 151.0 ppm (Cα) [89]. In crystals, the α-azido groups of triazides **55b** [102], **55c** [103], **55d** [95], **55e** [104], **55f** [105] and **55h** [100] show typical for aryl azides values of the C–Nα (~1.40 Å), Nα–Nβ (~1.26 Å) and Nβ–Nγ (~1.12 Å) bond distances and the C–Nα–Nβ (~113°) and Nα–Nβ–Nγ (~172°) angles. Almost the same values of the C–Nα (~1.39 Å), Nα–Nβ (~1.25 Å) and Nβ–Nγ (~1.12 Å) bond distances are observed as well in the γ-azido groups of triazides **55b**–**f** and **55h**. However, these groups have essentially larger the C–Nα–Nβ (~120°) angles and smaller the Nα–Nβ–Nγ (~169°) angles. In addition, the C–C–Nα–Nβ torsion angles of these groups vary from 2 (**55h**) till 40° (**55e**), depending on the bulkiness of the substituents in positions 3 and 5 of the pyridine ring. According to theory, the smaller the Nα–Nβ–Nγ angle and the larger the C–Nα–Nβ angle in azides, the higher the reactivity of azides in reactions with dipolarophiles, phosphines, phospites and reductans [105,106,107].

**Scheme 14:**
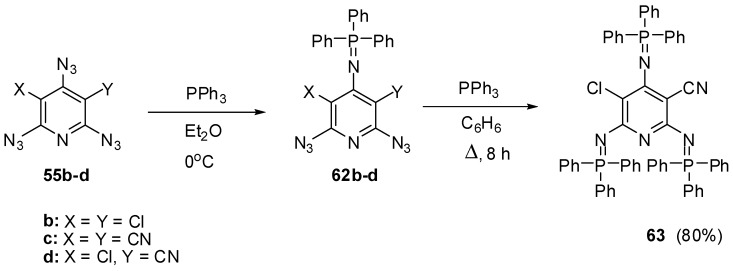
Selective Staudinger reactions of triazides **55b**–**d**.

Owing to the presence of nonequivalent azido groups on the pyridine ring, triazides **55a**–**d** were used as model compounds to investigate selective reactions on the azido groups of aromatic polyazides [34,35]. It was found that the Staudinger phosphorylation of triazides **55b**–**d** in ether at 0 °C occurs selectively on the most electron-deficient γ-azido groups to give iminophosphoranes **62b**–**d** in 93%–97% yields (Scheme 14) [93,108]. Similarly to preparation of *tris*-adduct **57** from triazide **55a** (Scheme 12), diazide **62c** reacted with an excess of triphenylphosphine on boiling in benzene to give *tris*-adduct **63** in 80% yield [93].

**Scheme 15:**
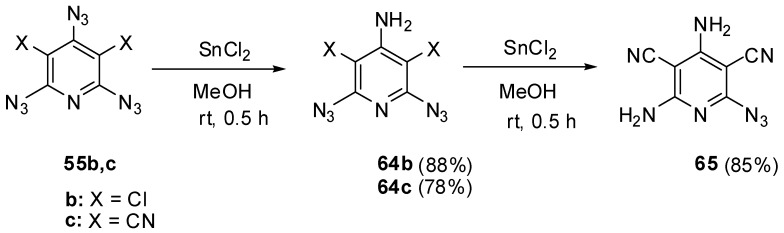
Selective reduction of triazides **55b**,**c**.

Beside selective phosphorylation, the γ-azido groups of triazides **55b**–**d** are selectively reduced to amines. Thus, the reactions of triazides **55b**,**c** with stannous chloride in methanol at room temperature afforded aminodiazides **64b**,**c** (Scheme 15) [109]. The more electron-deficient diazide **64c** was further reduced with stannous chloride in methanol at room temperature till diamine **65**.

**Scheme 16:**
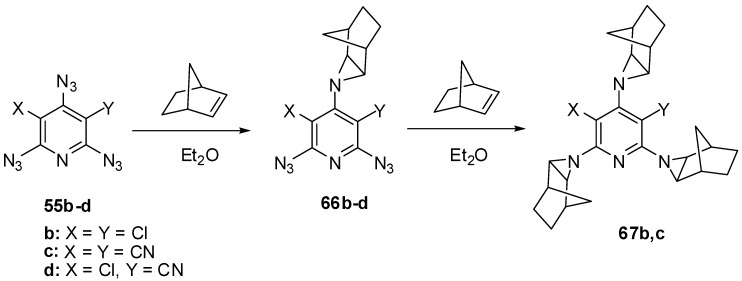
Selective addition of norbornene to triazides **55b**–**d**.

The electron-deficient γ-azido groups of triazides **55b**–**d** are also the most reactive toward electron-rich dipolarophiles [90,110]. Thus, the reaction of triazide **55c** with norbornene in ether at 0 °C occurred regio- and stereoselectively to give *exo*-adduct **66c** in high yield (Scheme 16) [110]. At room temperature, triazide **66c** readily reacted with an excess of norbornene to form *tris*-adduct **67c** in almost quantitative yield. Similar reactions of less electron-deficient triazides **55b**,**d** stopped at the stage of formation of *mono*-adducts **66b**,**d** [90,110]. The boiling of *mono*-adduct **66b** with norbornene in benzene gave *tris*-adduct **67b** in 56% yield [110]. On the other hand, the reactions of *mono*-adduct **66b** with norbornene in ether at room temperature in the presence of Rh_2_(OAc)_4_ as a catalyst gave *tris*-adduct **67b** in 90% yield [110].

**Scheme 17:**
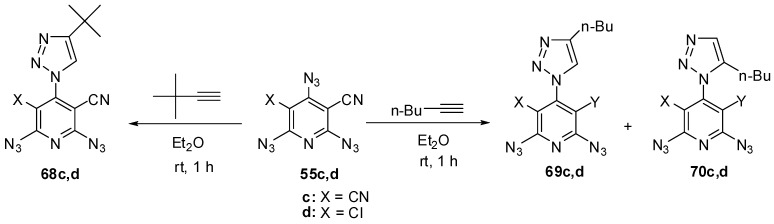
Selective addition of butylacetylenes to triazides **55c**,**d**.

The reactions of triazides **55c**,**d** with electron-rich *tert*-butylacetylene in ether at room temperature also occurred regioselectively on the γ-azido groups to form *mono*-adducts **68c**,**d** in ~85% yield (Scheme 17) [111]. Similar reactions of triazides **55c**,**d** with less sterically hindered *n*-butylacetylene gave mixtures of diazides **69c**,**d** (~82%) and **70c**,**d** (~8% yield) [111].

**Scheme 18:**
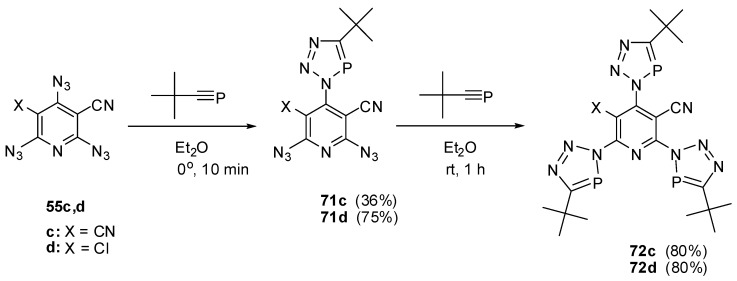
Selective addition of *tert*-butylphosphaacetylene to triazides **55c**,**d**.

In ether at 0 °C, the extremely reactive *tert*-butylphosphaacetylene reacted with triazides **55c**,**d** to form *mono*-adducts **71c**,**d** (Scheme 18) [91,112,113]. The latter reacted with an excess *tert*-butylphosphaacetylene in ether at room temperature to give *tris*-adducts **72c**,**d** [91,112,113].

**Scheme 19:**
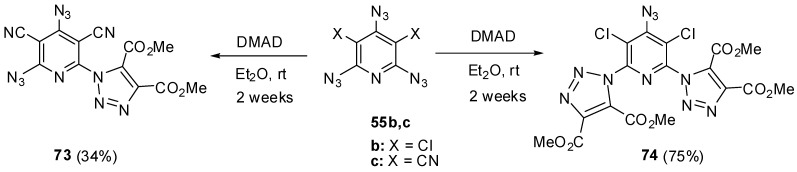
Reactions of triazides **55b**,**c** with dimethyl acetylenedicarboxylate.

Since high electron-deficiency, triazides **55a**–**j** are not much reactive toward electron-deficient dipolarophiles. The reaction of triazide **55c** with DMAD in ether at room temperature for 2 weeks occurred selectively on the least electron-deficient α-azido group to give triazole **73** in 34% yield (Scheme 19) [114]. Similar reaction of less electron-deficient triazide **55b** with DMAD led to the formation of bis-adduct **74** in 75% yield [114].

**Scheme 20:**
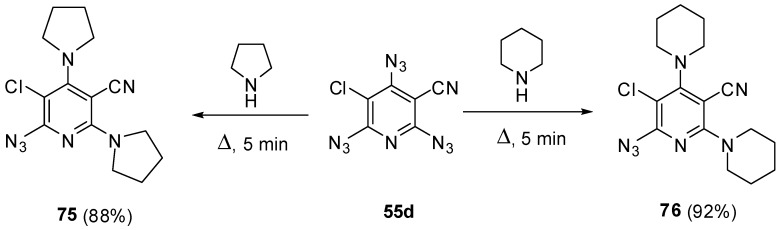
Reactions of triazide **55d** with aliphatic amines.

Owing to high electron-deficiency, triazides **55a**–**j** readily react with aliphatic amines. Thus, a brief (5 min) boiling of triazide **55d** in pyrrolidine or piperidine gave diamines **75** and **76** in high yields (Scheme 20) [115]. Most probably, these reactions occur by a radical mechanism involving electron transfer from amines to triazides followed by the collapse of diradical amine-azide pairs into aminopyridine and gaseous HN_3_. The reactions stop at the stage of *bis*-amination since low electron-deficiency of azides **75** and **76** [115]. Formally, these reactions are identical to amination of cyanuric triazide with aliphatic amines (see Scheme 2).

**Scheme 21:**
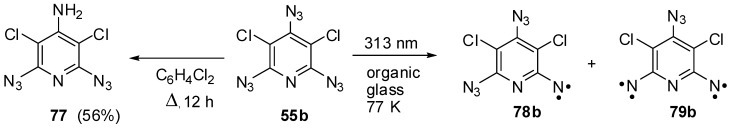
Selective thermolysis and photolysis of triazide **55b**.

On thermolysis, triazides **55a**–**j** undergo selective cleavage of the least durable N–N_2_ bonds of the γ-azido groups. Thus, the reflux of triazide **55b** in *p*-dichlorobenzene at 160 °C for 12 h gave aminodiazide **77** in 56% yield (Scheme 21) [116]. On the other hand, the photolysis of triazide **55b** in frozen at 77 K organic solutions occurred selectively on the α-azido groups to give triplet nitrene **78b** and quintet dinitrene **79b** as the major paramagnetic products (Scheme 21) [12,117]. This selectivity was explained by extremely low stability of the excited states of **55b** arising after local excitation of the α-azido groups [117].

**Scheme 22:**
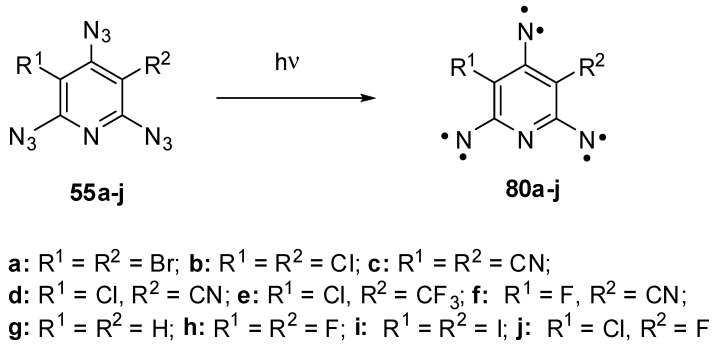
Photochemical generation of septet trinitrenes from triazides **55a**–**j**.

Triazides **55a**–**j** have played an important role in investigations of high-spin organic compounds. Before that, nothing was known about the UV-vis and IR spectra of organic hexaradicals with the ground septet spin-state. Even EPR spectra of septet trinitrenes were never reported. It was surmised that such trinitrenes should show the magnetic anisotropy of *D* ≈ −0.055 cm^−1^ [10,118]. The photolysis of triazides **55b** and **55h** in cryogenic matrices allowed the first registration of IR and UV-vis spectra of septet trinitrenes **80b** and **80d** (Scheme 22) [11,13]. It was found that these trinitrenes are photochemically very stable and do not rearrange into low-molecular-weight products. Moreover, high-spin nitrenes generated by the photolysis or γ-radiolysis of triazide **55b** were stable in crystals of the host triazide even at 230 K [102,103]. EPR studies of the photolysis of triazides **55a**–**j** showed that containing light atoms trinitrenes **55b**–**h** have nearly the same magnetic parameters of *D* ≈ −0.102 cm^−1^ and *E* ≈ 0.003 cm^−1^, independent on substituents in positions 3 and 5 of the pyridine ring [20,21,22,23,119]. Only trinitrene **80a** was an exception. Due to the presence of two heavy bromine atoms on the pyridine ring, this trinitrene showed the record value of |*D*| = 0.297 cm^−1^ among all organic septet hexaradicals [26]. Recent high-level *ab initio* calculations have proved that trinitrene **80a** has the negative sign of *D* and can be assigned to strong organic magnetic molecules [120]. The finding that heavy atoms drastically strengthen the magnetic properties of organic polyradicals opened up principally new prospects in design of organic magnetic materials.

## 6. Triazidobenzenes

**Scheme 23:**
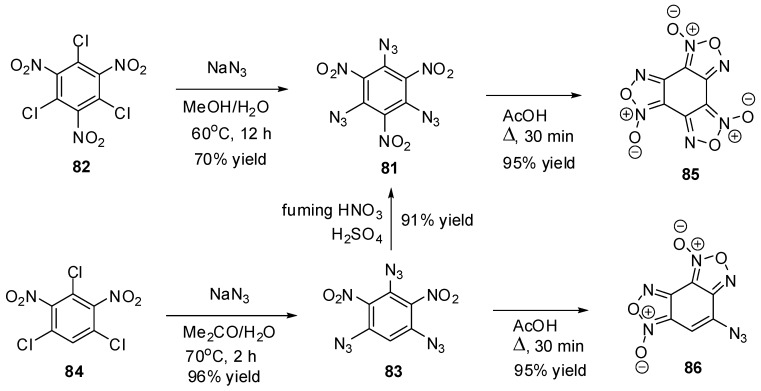
Synthesis of triazides **81** and **83**.

To the moment, there are known just nine triazidobenzenes. The first of them, 1,3,5-triazido-2,4,6-trinitrobenzene (**81**), was prepared by Turek in 1931, using the azidation of trichloride **82** with sodium azide in hot methanol (Scheme 23) [121]. Later, a more efficient way to preparation of **81**, based on nitration of triazide **83**, was developed [122,123,124]. The intermediate triazide **83** is readily obtained by refluxing trichloride **84** with sodium azide in the mixture of acetone and methanol [122]. Triazides **81** and **83** can also be obtained in 65%–90% yields by azidation of the corresponding chlorides **82** and **84** with sodium azide in aqueous acetone, methanol or dimethylsulfoxide solutions [125,126]. On boiling in acetic acid, both triazides **81** and **83** form benzofuroxans **85** and **86** in almost quantitative yields (Scheme 23) [122,126].

Triazides **81** and **83** are bright yellow solids with melting points of 107–108 and 128–130 °C, respectively [121,122,123,124,125,126]. Both triazides are typical high energy density organic materials that are rather sensitive toward impact and friction and very sensitive toward electrostatic discharge [74,124].

**Scheme 24:**
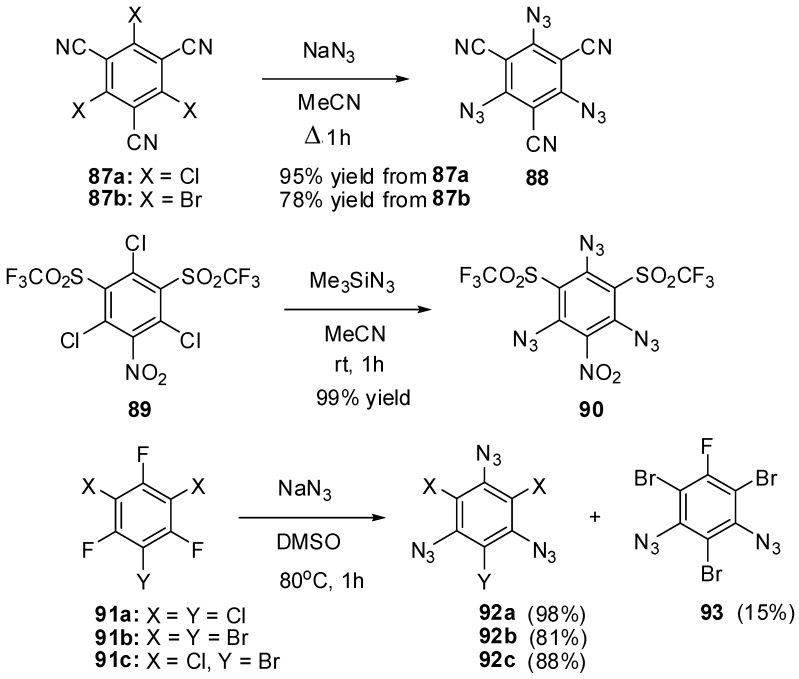
Synthesis of triazides **88**, **90** and **92a**–**c**.

The nucleophilic substitution reactions were also used to prepare triazidobenzenes **88**, **90** and **92a**–**c** from the corresponding halides (Scheme 24) [127,128,129,130]. The azidation of halides **87a**,**b** with sodium azide in boiling acetonitrile gave triazide **88** in high yield [127,128]. Similarly, the azidation of trichloride **89** with trimethylsilylazide in acetonitrile at room temperature afforded triazide **90** in quantitative yield [129]. Unfortunately, halides **87a**,**b** and **89** are hardly available compounds what seriously limits the use of triazides **88** and **90** in practice. Thus, tribromide **87b** is prepared from mesitylene in six steps [128]. Both triazides **88** (m.p. 161 °C) and **90** (m.p. 113–114 °C) are solids that are almost insensitive toward impact, friction and electrostatic discharge. Triazide **88** has the highest melting point among all six-membered aromatic triazides [128]. Recently, a simple and efficient method of preparation of triazidobenzenes **92a**–**c** from commercially available trifluorides **91a**–**c** has been developed (Scheme 24) [130]. Trifluorides **91a** and **91c** readily underwent chemoselective defluorination on heating in dimethylsulfoxide solutions with sodium azide to form triazides **92a** and **92c** in high yields. However, a similar reaction of trifluoride **91b**, containing the least electron-withdrawing substituents on the benzene ring, stopped at the stage of formation of a mixture of diazide **93** and triazide **92b**. The latter was obtained in 81% yield by refluxing the mixture of **93** and **92b** with sodium azide in aqueous acetone, from which triazide **92b** precipitated. All triazides **92a** (m.p. 94 °C), **92b** (m.p. 105 °C) and **92c** (m.p. 83 °C) are colorless solids that are insensitive toward impact, friction and electrostatic discharge. Owing to their simple and efficient preparation from commercially available starting materials, triazides **92a**–**c** may be of considerable interest as cross-linking agents for polymer chemistry and as starting compounds in synthetic chemistry.

**Scheme 25:**
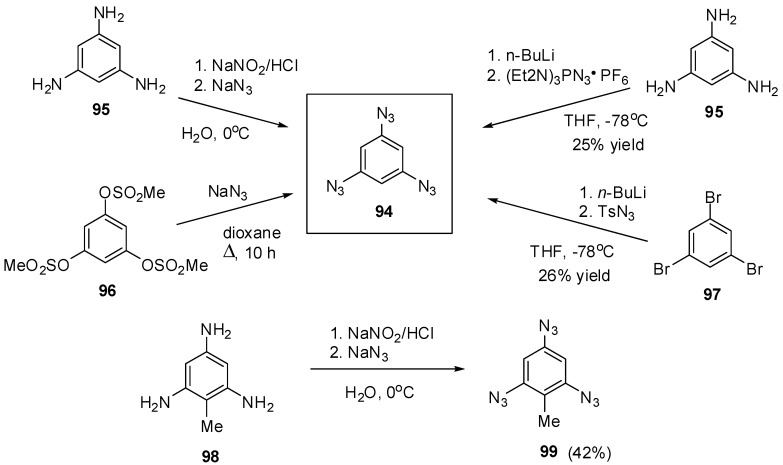
Synthesis of triazides **94** and **99**.

**Scheme 26:**
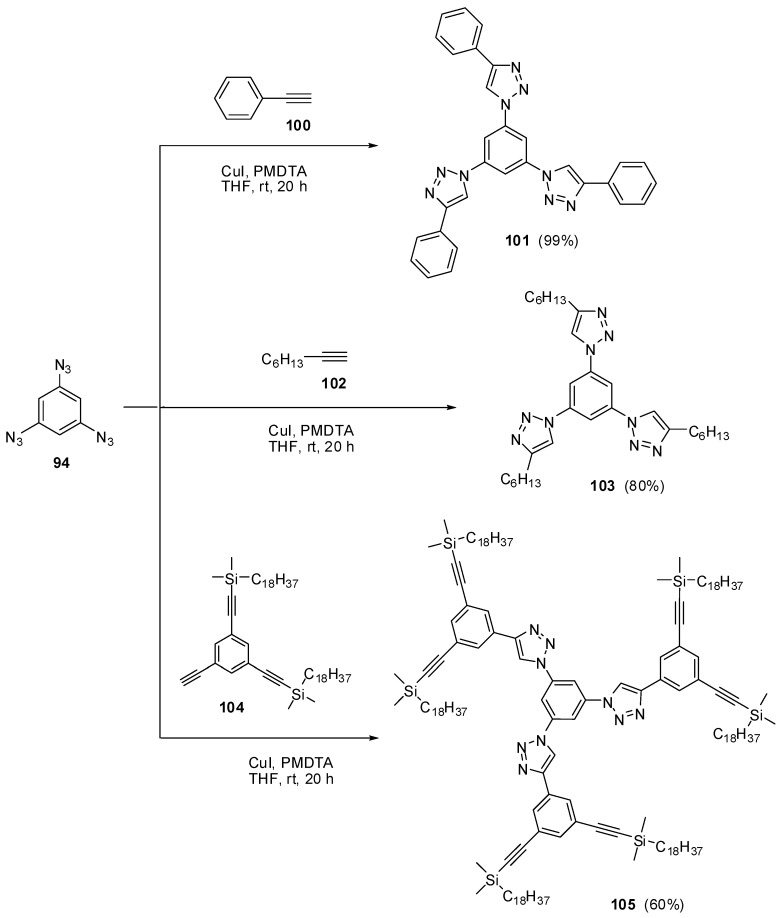
Some click-reactions of triazide **94**.

**Scheme 27:**
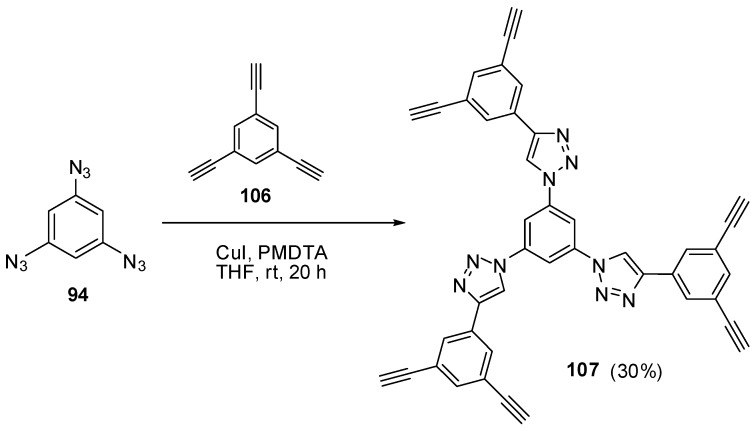
Сlick-reaction of triazide **94** with *tris*-acetylene **106**.

The most contradictory data were reported on the synthesis and properties of triazide **94**. In 1967, Breslow and Marcantonio patented the preparation of **94**, based on diazotization of triamine **95** (Scheme 25) [131]. However, neither the protocol of preparation nor the yield and melting point of **94** were reported. Later, Kim and Lee have described the preparation of **94** by azidation of ester **96** in boiling dioxane [132]. Again, neither the yield nor melting point of **94** was reported. It was just mentioned that NMR spectra of triazide **94** display a signal of protons at δ 7.30 ppm and a signal of carbons at δ 128.0 ppm. Very recently, the synthesis of triazide **94** from various precursors has been investigated by Juriček [133]. He found that triamine **95** is very unstable in acidic media and cannot be converted in triazide **94**, using the diazotization reaction. The highest yield (26%) of triazide **94** was achieved in the reaction of tribromobenzene **97** with *n*-butyllithium followed by the diazo-transfer reaction from tosylazide to intermediate trilithiobenzene (Scheme 25). The stepwise treatment of triamine **95** at first with *n*-butyllithium and then with (Et_2_N)_3_PN_3_·PF_6_ salt as a diazo-transfer reagent gave triazide **94** in 25% yield (Scheme 25) [133]. Triazide **94** was obtained as a yellow solid that was insensitive toward impact and friction. NMR spectra of triazide **94** displayed a signal of protons at δ 6.45 ppm and two signals of carbon atoms at δ 106.1 (CH) and 143.2 (C–N_3_) ppm. These spectroscopic characteristics agreed well with ^1^H- and ^13^C-NMR spectra of triazide **99** obtained in 42% yield by diazotization of triamine **98** (Scheme 25) [24]. Similarly to triazide **94**, triazide **99** was obtained as a yellow solid with a melting point of 103–104 °C. NMR spectra of triazide **99** displayed two signals of protons at δ 1.93 (Me) and 6.48 (CH) ppm and four signals of carbon atoms at δ 10.0 (Me), 103.8 (CH), 117.2 (C–Me), 138.4 (C_4_–N_3_) and 140.3 (C_2,6_–N_3_) ppm. Similarly to triazide **94**, triazide **99** is also insensitive toward impact and friction. Both triazides **94** and **99** are efficient cross-linking agents for polymer chemistry [131,134]. These triazides are also promising starting materials for supramolecular chemistry. Thus, the copper-catalyzed azide-alkyne cycloadditions (CuAAC- or click-reactions) of triazide **94** with acetylenes **100**, **102** and **104** afforded *tris*-adducts **101**, **103** and **105** (Scheme 26) [133]. The more unpredictable reaction of triazide **94** with *tris*-acetylene **106** gave *tris*-adduct **107** in just 30% yield (Scheme 27) [133]. The latter can be obtained in 99% yield by alkali hydrolysis of *tris*-adduct **105** [133]. Both hexaacetylenes **105** and **107** possess a huge synthetic potential and are of considerable interest as building-blocks for synthetic chemistry.

Recent X-ray diffraction studies have shown that the azido groups of triazides **81** and **88** are rotated out of the C_6_ plane in order to reduce steric repulsion [124,128]. Due to this effect, all three azido groups of **81** and **88** are structurally nonequivalent and slightly differ in the C–Nα, Nα–Nβ and Nβ–Nγ bond distances and the C–Nα–Nβ and Nα–Nβ–Nγ angles. These parameters of triazides **81** and **88** are very close to that of the γ-azido groups of triazidopyridines **55b** and **55c** [102,103,104]. It is therefore not surprising that the γ-azido groups of triazidopyridines **55b** and **55c** and the azido groups of triazides **92a** and **92b** display similar chemical shifts in ^15^N-NMR spectra [73,130]. In ^13^C-NMR spectra of *D*_3h_ symmetric triazides **88**, **92a**, **92b** and **94**, the signals of the C–N_3_ atoms are the most deshielded and manifested in the δ 134–150 ppm region, while the signals of the C–R atoms are usually observed in the δ 95–122 ppm region [128,130,133].

**Scheme 28:**
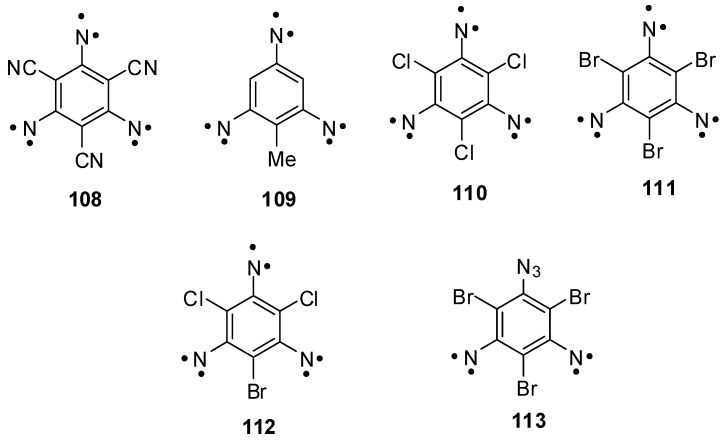
High-spin nitrenes obtained by photolysis of triazides **88**, **92a**–**c** and **99**.

Triazides **88**, **92a**–**c** and **99** have played a historical role in investigations of high-spin organic molecules. Trinitrene **108** obtained by the photolysis of triazide **88** in frozen organic solutions became the first organic septet hexaradical that was detected with X-band EPR spectroscopy (Scheme 28) [10]. Based on intuitive assignment of signals in EPR spectrum, the magnetic parameter *D* ≈ −0.055 cm^−1^ of trinitrene **108** was calculated. For many years, this value of *D* served as a benchmark in investigations of many other high-spin organic molecules [117]. Only recently, owing to the appearance of modern computer line-shape EPR spectral simulation programs, it was established that trinitrene **108** has *D* = −0.092 cm^−1^ [29]. Similar values of *D* were also determined for trinitrenes **109** (*D* = −0.0934 cm^−1^) and **110** (*D* = −0.0957 cm^−1^) obtained by the photolysis of triazides **99** and **92a**, respectively [24,29]. These studies showed that septet 1,3,5-trinitrenobenzenes have relatively low spin densities on the nitrene units and, as a result, relatively weak dipolar spin-spin interactions and magnetic properties among septet trinitrenes. On the other hand, the introduction of heavy atoms in positions 2, 4 and 6 of septet 1,3,5-trinitrenobenzenes allows the preparation of organic hexaradicals possessing the record values of magnetic anisotropy. Thus, trinitrene **111** obtained by the photolysis of triazide **92b** shows *D* = −0.203 cm^−1^ [33]. In such molecules, the large negative value of *D* arises due to very strong anisotropic spin-orbit interactions. As a result, the *D* value of trinitrene **111** exceeds almost two times the *D* value of septet 2,4,6-trinitreno-s-triazine **17** (see Scheme 5) possessing the strongest dipolar spin-spin interactions among septet trinitrenes. Photochemical studies of triazidobenzenes provide also important information about the effect of subtle structural changes in the molecules of these compounds on magnetic properties of organic polyradicals. Thus, it was found that, in contrast to trinitrenes **108**, **109**, **110** and **111**, trinitrene **112** has a positive sign of *D* = 0.124 cm^−1^ and does not possess the magnetism [32]. Since the presence of only one heavy bromine atom in the molecule, the principal magnetic Z-axis of trinitrene **112** lies in the molecular plane perpendicularly to the C–Br bond, and the principal values *D*_xx_, *D*_yy_ and *D*_zz_ of the total tensor **D** have such magnitudes and signs for which the total parameter *D* is large in magnitude and positive in sign. Another unexpected result was obtained during W-band EPR studies of quintet dinitrene **113** formed in the photolysis of triazide **92b** [33]. In contrast to all known quintet dinitrenes, dinitrene **113** showed negative sign of *D* = −0.306 cm^−1^ due to the effect of three heavy bromine atoms.

## 7. Tetraazides

**Scheme 29:**
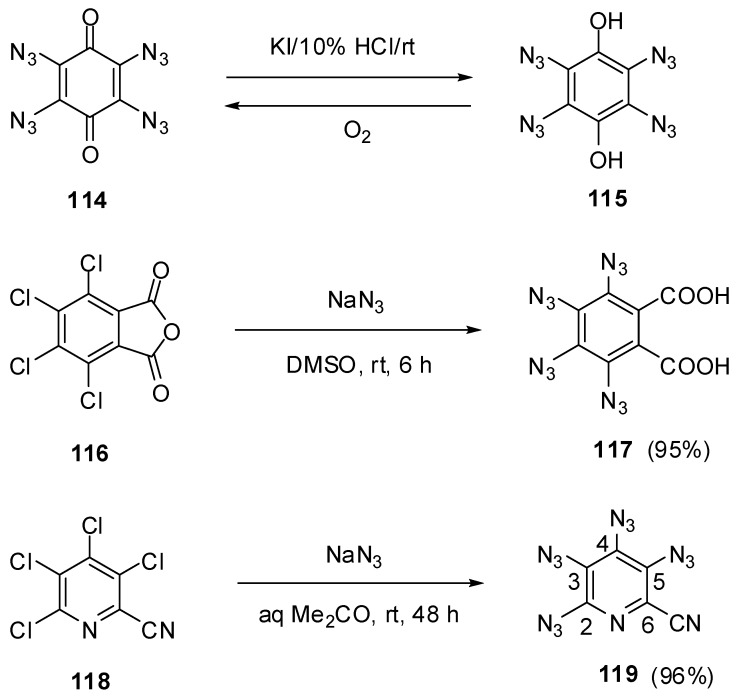
Synthesis of tetraazides **115**, **117** and **119**.

Six-membered aromatic tetraazides represent one of the least studied classes of organic compounds. To the moment, the synthesis of only four such tetraazides has been reported, and only one of them was characterized with modern spectroscopic methods. The first representative of six-membered aromatic tetraazides, 1,2,4,5-tetrazido-3,6-dihydroxybenzene (**115**), was prepared by Sorm in 1939, using the reduction of tetraazidoquinone **114** with potassium iodide in acidic media (Scheme 29) [135]. During this reaction, deep-blue quinone **114** was turned into hydroquinone **115** as insoluble in water white solid. The latter was metastable in the air and quickly oxidized to the starting quinone **114**. On comparison with very sensitive tetraazidoquinone **114**, tetraazidohydroquinone **115** was much less sensitive to impact, friction and spark. The second representative of this class of compounds, tetrazidophthalic acid **117**, was prepared in 95% yield by the reaction of tetrachlorophthalic anhydride **116** with sodium azide in dimethylsulfoxide at room temperature (Scheme 29) [136]. Tetraazide **117** was obtained as a white solid with a melting point of 113 °C. Since its very high sensitivity to mechanical stimuli, no any further studies of **117** were carried out. Finally, Pannell in 1975 has reported the synthesis of tetraazide **119** by the reaction of tetrachloride **118** with sodium azide in dimethylformamide at 80 °C (Scheme 29) [137,138]. Recently, this tetraazide was obtained by azidation of tetrachloride **118** with sodium azide in aqueous acetone at room temperature in 96% yield [91].

On crystallization from ethanol, tetraazide **119** is obtained as a brown solid with a melting point of 103 °C [92]. This tetraazide is very sensitive to impact and friction and violently explodes on quick heating at 23 °C. On working with it, one should always handle **119** only with plastic spatulas and use thick gloves behind a blast shield. Due to interatomic interactions of the neighboring azido groups in the molecule, the rate of the thermal decomposition of **119** is 1000 times higher than that of 2,4,6-triazido-3,5-dicyanopyridine (**55c**) [139]. Most likely, the thermal decomposition of **119** follows the chain-mechanism, yielding molecular nitrogen and C_3_N_4_ carbon nitrides as the final products. The IR and UV spectra of tetraazide **119** are very similar to those of triazide **55c** [92]. The ^13^C-NMR spectrum of tetraazide **119** shows six signals of the carbon atoms at δ 113.4 (CN), 118.2 (C-6), 122.8 (C-3), 129.7 (C-5), 133.1 (C-4) and 145.1 ppm (C-2) [91].

**Scheme 30:**
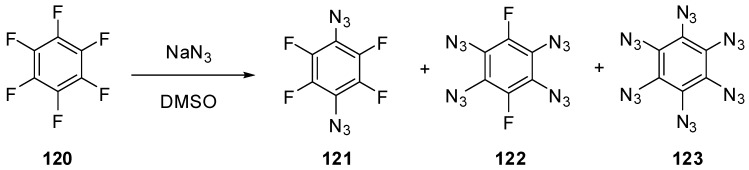
Azidation of hexafluorobenzene **120**.

To the moment, nothing is known about the properties of six-membered aromatic compounds with five and six azido groups in the ring. It has just been reported that azidation of hexafluorobenzene (**120**) with sodium azide in hot dimethylsulfoxide led to the formation of a mixture of diazide **121** and tetraazide **122** as the major products as well as hexaazide **123** as a minor product (Scheme 30) [140]. However, during the next five decades, no any other information on the synthesis, properties and use of tetraazide **122** and hexaazide **123** appeared in the literature. So far, hexaazide **123** as well as pentaazidopyridine and tetraazidopyrimidine can be considered only as hypothetical molecules in theoretical studies [141,142,143].

## 8. Summary

Of the twenty five six-membered aromatic triazides known to date, eighteen have been synthesized and spectrally characterized only in the last two decades. Many of these triazides are prepared in one step and in high yield from commercially available starting halides. Moreover, most of these triazides are rather safety compounds and can explode only under very specific conditions. Owing to these features, six-membered aromatic triazides may be of considerable interest as starting materials for synthetic chemistry and photochemistry as well as cross-linking agents for polymer chemistry and microelectronics. The high antitumor activity of 2,4,6-triazidopyrimidine against Sarcoma 180, Piss lymphosarcoma and Guerin carcinoma indicates that some of aromatic triazides may possess interesting biological properties. However, the most promising application of aromatic triazides may be their use as starting materials in click-chemistry aimed at the design of new polyfunctional supramolecular systems possessing useful chemical, physical and biological properties. Thus, for instance, just the successive cycloaddition of different acetylenes to such triazides already provides unique opportunities to synthesize a great variety of new organic compounds, using relatively simple experimental procedures.
